# Peripheral natural killer cells and myeloid-derived suppressor cells correlate with anti-PD-1 responses in non-small cell lung cancer

**DOI:** 10.1038/s41598-020-65666-x

**Published:** 2020-06-03

**Authors:** Je-In Youn, Su-Myeong Park, Seyeon Park, Gamin Kim, Hee-Jae Lee, Jimin Son, Min Hee Hong, Aziz Ghaderpour, Bumseo Baik, Jahirul Islam, Ji-Woong Choi, Eun-Young Lee, Hang-Rae Kim, Sang-Uk Seo, Soonmyung Paik, Hong In Yoon, Inkyung Jung, Chun-Feng Xin, Hyun-Tak Jin, Byoung Chul Cho, Seung-Yong Seong, Sang-Jun Ha, Hye Ryun Kim

**Affiliations:** 1 Department of Biomedical Sciences, Seoul National University College of Medicine, Seoul, Korea; 2 Department of Biochemistry, College of Life Science & Biotechnology, Yonsei University, Seoul, Korea; 3 Wide River Institute of Immunology, Seoul National University College of Medicine, Hongcheon, Korea; 4 Research Institute, ProGen, Inc., Seongnam-si, Gyeonggi-do Korea; 5 Yonsei Cancer Center, Division of Medical Oncology, Yonsei University College of Medicine, Seoul, Korea; 6 Department of Anatomy and Cell Biology, Seoul National University College of Medicine, Seoul, Korea; 7 Severance Biomedical Science Institute, Yonsei University College of Medicine, Seoul, Korea; 8 Yonsei Cancer Center, Department of Radiation Oncology, Yonsei University College of Medicine, Seoul, Korea; 9 Department of Biostatistics and Medical Informatics, Yonsei University College of Medicine, Seoul, Korea; 10 JE-UK Institute for Cancer Research, JEUK Co., Ltd., Gumi-City, Kyungbuk Korea; 11 Department of Microbiology and Immunology, Seoul National University College of Medicine, Seoul, Korea

**Keywords:** Lung cancer, Immunotherapy, Tumour immunology

## Abstract

Inhibition of immune checkpoint proteins like programmed death 1 (PD-1) is a promising therapeutic approach for several cancers, including non-small cell lung cancer (NSCLC). Although PD-1 ligand (PD-L1) expression is used to predict anti-PD-1 therapy responses in NSCLC, its accuracy is relatively less. Therefore, we sought to identify a more accurate predictive blood biomarker for evaluating anti-PD-1 response. We evaluated the frequencies of T cells, B cells, natural killer (NK) cells, polymorphonuclear myeloid-derived suppressor cells (PMN-MDSCs), mononuclear myeloid-derived suppressor cells (M-MDSCs), and Lox-1^+^ PMN-MDSCs in peripheral blood samples of 62 NSCLC patients before and after nivolumab treatment. Correlation of immune-cell population frequencies with treatment response, progression-free survival, and overall survival was also determined. After the first treatment, the median NK cell percentage was significantly higher in responders than in non-responders, while the median Lox-1+ PMN-MDSC percentage showed the opposite trend. NK cell frequencies significantly increased in responders but not in non-responders. NK cell frequency inversely correlated with that of Lox-1^+^ PMN-MDSCs after the first treatment cycle. The NK cell-to-Lox-1^+^ PMN-MDSC ratio (NMR) was significantly higher in responders than in non-responders. Patients with NMRs ≥ 5.75 after the first cycle had significantly higher objective response rates and longer progression-free and overall survival than those with NMRs <5.75. NMR shows promise as an early predictor of response to further anti-PD-1 therapy.

## Introduction

Non-small cell lung cancer (NSCLC) is the leading cause of cancer-related mortality worldwide^1^. Recently, immune checkpoint inhibitors (ICIs) have demonstrated remarkable efficacy in the treatment of NSCLC with substantial improvements in survival^2,3^. However, response rates to anti-programmed death 1 (PD-1) therapy remain relatively low (15–40%)^4^, thus there is an increasing demand to identify predictive biomarkers to identify patients who will benefit from this treatment. Currently, tumour PD-1 ligand (PD-L1) expression determined by immunohistochemistry (IHC) is the only accepted predictive biomarker for anti-PD-1 therapy response in clinical practice; however, its prediction accuracy is relatively weak. For example, the Checkmate 017 and 057 trials revealed that some PD-L1-negative patients could, in fact, benefit from anti-PD-1 therapy, and *vice versa*^5,6^. Recently, CD8^+^ T-cell infiltration, the inflammatory tumour signature, T-cell receptor clonality, and the somatic mutational burden have all shown promise as alternative predictive biomarkers for anti-PD-1 therapy^7,8^, but to date, none has been demonstrated to be a useful biomarker for patient selection.

A predictive biomarker found in peripheral blood or serum would be ideal for clinical application as a non-invasive and simple method. Thus, the identification of such biomarkers would significantly improve therapeutic success and clinical management. Nevertheless, there are sparse data available on the efficacy of such markers for predicting the outcome of ICI therapy in patients with NSCLC, especially with respect to anti-PD-1 therapy^9,10^. Low CD62L^low^ CD4^+^ T-cell frequency and high neutrophils to lymphocytes ratio (NLR) respectively correlated with poor survival in patients with NSCLC^11,12^. Recently, our group reported that peripheral frequencies of two representative immune suppressive cell populations in cancer, regulatory T cells and myeloid-derived suppressor cells (MDSCs), were correlated with response to anti-PD-1 therapy in patients with NSCLC^13^. Natural killer (NK) cells are innate lymphocytes with anti-cancer cytotoxic activity; they also maintain balance in the immune response by releasing cytokines and chemokines^14^. Anti-PD-1 therapy has been shown to induce a potent NK cell response, considered to be indispensable for the anti-tumour efficacy. Accordingly, NK cells, along with T cells, might contribute to prediction of the anti-PD-1 therapy response in cancer patients^14^.

In the present study, we further analysed the frequencies of various immune cell populations including NK cells and MDSC subsets in the peripheral blood of patients with NSCLC pre- and post-nivolumab and evaluated their relation to clinical outcome, with the goal of identifying improved immune markers for patient selection for PD-1 inhibitors.

## Results

### Patient characteristics and clinical outcomes

The baseline characteristics of 62 patients with NSCLC treated with nivolumab are summarised in Table 1. The median age was 63 years. The majority of patients were male, showed an ECOG performance status of 0 or 1, were smokers, had adenocarcinoma, and had previously received one or two lines of treatment. The median duration of nivolumab administration was 42 days (range 8–740 days), and the ORRs were 36%. With a median follow-up of 165 days, the median progression free survival (PFS) and overall survival (OS) values were 43 (range 8–740) days and 173 (range 13–901) days, respectively.

**Table 1:** Clinical characteristics of patients treated with nivolumab.

Variables	Patients (*n* = 62) *n* (%)
**Age, years**	
Median (range)	63 (39–87)
**Sex**	
Men	40 (64.5)
Women	22 (35.5)
**ECOG performance status**	
0	9 (14.5)
1	43 (69.4)
2	10 (16.1)
**Smoking**	
Never/light smoker	23 (37.1)
Smoker	39 (62.9)
**Histology**	
Adenocarcinoma	47 (75.8)
Squamous cell carcinoma	15 (24.2)
**Mutation type**	
*EGFR* mutation	7 (11.3)
*ALK* or *ROS1* rearrangement	1 (1.6)
Wild type	54 (87.1)
**Previous treatment**	
Chemotherapy	35 (56.4)
Targeted therapy	9 (14.5)
Immunotherapy	0 (0)
Surgery	4 (6.4)
Radiotherapy	7 (11.2)
**No. of prior therapies**	
1	29 (46.8)
2	12 (19.4)
>2	21 (33.8)

### Immune-cell frequencies differ between Nivolumab responders and non-responders after treatment

**Figure 1:**
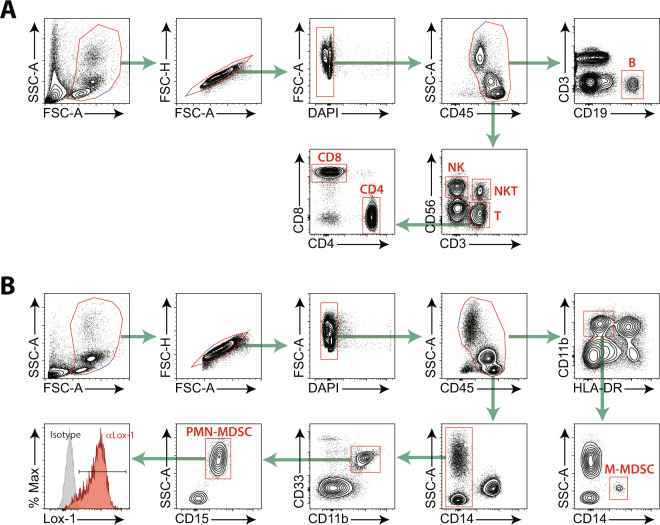
Gating strategies for peripheral blood immune cells. (**A**) Strategies for lymphocytes: CD19^+^ B cells, CD56^+^NK cells, CD3^+^CD56^+^NKT cells, CD3^+^ total T cells, CD3^+^CD4^+^ T cells, and CD3^+^CD8^+^ T cells. (**B**) Strategies for MDSCs: HLA-DR^-/low^CD11b^+^CD14^+^ M-MDSCs, CD14^-^CD11b^+^CD33^+^CD15^+^ PMN-MDSCs, and Lox-1^+^ PMN-MDSCs. Singlet cells were selected and dead cells were removed based on the scatter plot.

To determine the effect of anti-PD-1 therapy on immune cells, we monitored T cells, B cells, NK cells, monocytes, and MDSCs in the peripheral blood of patients with advanced NSCLC both before and after the first round of nivolumab therapy. We also monitored the proportions of the M-MDSC and PMN-MDSC subsets as well as the expression of lectin-type oxidised low-density lipoprotein receptor 1 (Lox-1), which distinguishes between PMN-MDSCs and neutrophils (Fig. 1)^12^.

**Figure 2:**
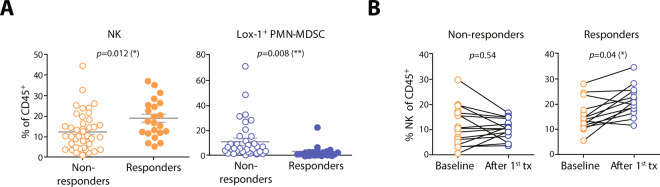
(**A**) Percentages of NK cells and Lox-1^+^ PMN-MDSCs among CD45^+^ T cells in non-responders and responders at 2 weeks after the first round of nivolumab. Dot plots represent frequencies of immune cells, and small horizontal lines indicate means (±SD). (**B**) Changes in NK frequencies between baseline and after the first nivolumab treatment in non-responders and responders. Each dot indicates a single patient. *P < 0.05, **P < 0.01 (two-tailed Student’s *t-*test).

At baseline, there were no significant differences in the frequencies of the tested immune cells between responders and non-responders (Supplementary Fig. 1). After the first treatment, the median percentage of NK cells was higher in responders, whereas the median percentage of Lox-1^+^ PMN-MDSCs in the responders was higher than that in the non-responders (Fig. 2A). There was a significant increase in the NK cell frequency after the first treatment in the responders but not in the non-responders (Fig. 2B). However, there were no significant differences in frequencies of CD4^+^ T, CD8^+^ T, CD19^+^ B, NKT cells, CD14^+^ monocytes or NLR (Supplementary Fig. 1).

### NK cell-to-Lox-1+ PMN-MDSC ratio (NMR) predicts early clinical responses to anti-PD-1 therapy

**Figure 3:**
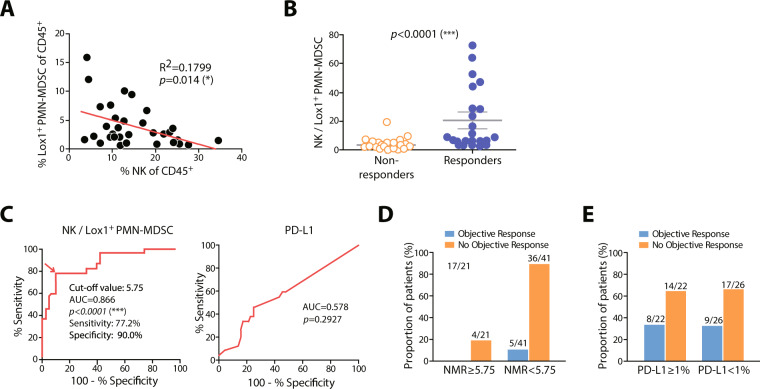
(**A**) Inverse correlation between Lox-1^+^ PMN-MDSCs and NK cells among CD45^+^ cells. (**B**) NK/Lox-1^+^ PMN-MDSCs ratios (NMRs) in non-responders and responders after the first round of treatment. (**C**) Receiver operating characteristic curves for nivolumab responses with respect to NMR or PD-L1. Proportions of responders and non-responders to nivolumab therapy according to (**D**) the NMR cut-off value of 5.75 and (**E**) PD-L1 positivity.

Intriguingly, we found an inverse correlation between the percentage of NK cells and Lox-1^+^ PMN-MDSC after the first nivolumab treatment (Fig. 3A). To derive an optimised cut-off value for distinguishing between responders and non-responders, we assessed the NMR, which was significantly higher in responders than that in non-responders after the first nivolumab treatment (Fig. 3B).

To further evaluate the performance of NMR as a predictive marker, we compared the prediction rate of NMR with that of PD-L1 expression, the currently used biomarker for PD-1 blockade. Of the 62 patients, archival tissue samples for 48 were available for assessment of PD-L1 expression by IHC. Twenty-two of these patients were PD-L1-positive (defined as ≥ 1% PD-L1^+^ tumour cells). Receiver operating characteristic curves (ROCs) for nivolumab response using NMR demonstrated that this parameter had high discriminatory ability. The area under the ROC curve (AUC) value was 0.866 [95% confidence interval (CI) 0.770–0.962, *P* < 0.0001], whereas the AUC value for PD-L1 IHC was 0.578 (95% CI 0.432–0.723, *P* = 0.2927; Fig. 3C). The optimised cut-off value of NMR was determined to be 5.75 with sensitivity of 77.2% (95% CI 0.546–0.922) and specificity of 90% (95% CI 0.763–0.972). The positive predictive value (i.e., response rate above the cut-off) of an NMR of 5.75 was 80.9% (17/21), whereas that of a PD-L1-positive rate ≥ 1% was 36.3% (8/22). The negative predictive value (i.e., the non-response rate below the cut-off) of the NMR was 87.8% (36/41; Fig. 3D), and that of PD-L1 was 65.4% (17/26; Fig. 3E). Thus, in some patients, the peripheral blood NMR cut-off value of 5.75 is a more reliable predictor of the response to nivolumab than PD-L1 levels in tumour tissues.

**Figure 4:**
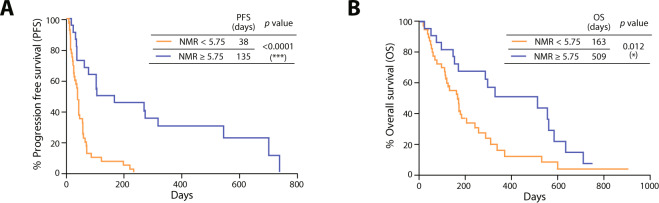
Progression-free survival (PFS) (**A**) and overall survival (OS) (**B**) according to the cut-off NMR value of 5.75. *P < 0.05, ***P < 0.001 (Mantel-Cox log-rank test).

Using the cut-off NMR value of 5.75, Kaplan-Meier survival analysis showed that patients with an NMR ≥ 5.75 after the first nivolumab treatment showed significantly longer median PFS (135 *vs*. 38 days; *P* < 0.0001) than did those with an NMR < 5.75 (Fig. 4A). The median OS (509 *vs*. 163 days; *P* = 0.012) of patients with an NMR ≥ 5.75 was also significantly longer than that of those with an NMR < 5.75 (Fig. 4B).

Moreover, in Cox regression models adjusted for age, sex, *EGFR* mutation, and PD-L1 expression, the adjusted hazard ratios (AHRs) for the risk of progression and OS after anti-PD-1 therapy were significant in patients with an NMR ≥ 5.75 (Table 2). Taken together, these data suggest that NMR after the first cycle of anti-PD-1 therapy strongly correlated with treatment outcomes, including ORR, PFS, and OS, in NSCLC patients.

**Table 2:** Factors affecting the progression-free survival and overall survival in patients after anti-PD-1 therapy based on multivariate analysis.

Variable	Category	Multivariate survival analysis Progression-free survival			Multivariate survival analysis Overall survival		
		AHR	95% CI	P	AHR	95% CI	P
Age (years)	≥65 vs. <65	1.273	0.709–2.284	0.419	1.147	0.629–2.092	0.655
Sex	Women vs. Men	0.812	0.428–1.540	0.523	1.127	0.569–2.233	0.732
EGFR status	Mutant vs. wild-type	2.158	0.954–4.882	0.065	2.563	1.083–6.063	0.032
PD-L1	≥ 1% vs. <1%	0.878	0.505–1.528	0.646	0.689	0.375–1.264	0.228
NMR	≥ 5.75% vs. <5.75	0.198	0.095–0.413	0.0001	0.369	0.190–0.719	0.003

Abbreviations: EGFR, epidermal growth factor receptor; NMR, NK cells to Lox-1^+^PMN-MDSC ratio; AHR, adjusted hazard ratio; CI, confidence interval.

## Discussion

In this study, we demonstrated that two types of immune cells, NK cells, and Lox-1^+^ PMN-MDSCs show a rapid response to anti-PD-1 therapy. After the first round of therapy, the frequency of NK cells was significantly higher in the responders than in the non-responders, whereas that of Lox-1^+^ PMN-MDSCs showed the opposite pattern. The NMR was significantly higher in responders than in non-responders, and the NMR after the first round of nivolumab therapy strongly correlated with treatment outcomes, including response, PFS, and OS, in patients with NSCLC.

NK cells are a crucial component of the immune surveillance system against cancer. They act by killing tumour cells *via* engagement of death receptors, secreting granzymes/perforins, and antibody-dependent cell-mediated cytotoxicity^15^. Recent studies have demonstrated that NK cells also play pivotal roles in cancer immunotherapy. When NK cells were depleted in mice, PD-1/PD-L1 blockade was completely ineffective^14^. In addition, the anti-tumour activity of NK cells was inhibited by PD-1/PD-L1 interactions and was restored by PD-1/PD-L1 blockade. Another immune-checkpoint molecule, the T cell immunoglobulin and immunoreceptor tyrosine-based inhibitory motif domain (TIGIT), was shown to mediate NK cell exhaustion in cancer, with the blockade of TIGIT restoring the anti-tumour activity of NK cells^16^. Moreover, TIGIT inhibition promoted tumour-specific T cell immunity and enhanced the survival of tumour-bearing mice, depending on the presence of NK cells. An increased frequency of NK cells has generally been correlated with an improvement in the OS of patients^17^. Recent clinical studies have demonstrated the contribution of NK cells in cancer patients treated with ICI. In patients with NSCLC treated with ICI, an allelic variant of the NK-cell receptor was associated with the NK-cell antitumor activity^18^. In metastatic melanoma, the frequencies of both intratumoral stimulatory dendritic cells and NK cells correlated with responsiveness to anti-PD-1 therapy^19^.

MDSCs are one of the main factors in creating an immune-suppressive microenvironment in tumours and thus contribute to tumour progression, angiogenesis, and metastasis^20,21^. Recently, an increase in MDSC frequency in the blood of patients with melanoma or NSCLC was suggested to serve as a useful prognostic biomarker for inadequate response to anti-PD-1 therapy^13,22^. Recent studies also suggested that the frequency of classical monocytes (>19.4%) and NLR before anti-PD1 therapy predicted favourable responses and survival^13,14^. However, in this study, the monocyte count did not identify responders among patients with NSCLC. A possible reason for this discrepancy is that different immune cells play essential roles in melanoma and NSCLC. MDSCs consist of two principal subsets: PMN-MDSCs and M-MDSCs. Interestingly, M-MDSCs are the major subset in melanoma patients, whereas PMN-MDSCs are dominant in patients with NSCLC^23^. Therefore, certain cancer types showing high expansion of PMN-MDSCs, including head and neck, breast, and pancreatic cancers, should be assessed while conducting further biomarker studies using the NMR for anti-PD-1 therapy.

PD-L1 expression in tumour cells, as determined by IHC is currently the only approved predictive biomarker for anti-PD-1 therapy. However, the accuracy of this test is relatively poor, making it difficult to discriminate between patients who would or would not benefit from anti-PD-1 therapy. Moreover, considering the low accessibility of tumour tissues from patients and tumour heterogeneity depending on the biopsy site, the examination of peripheral blood should be a much simpler and more consistent method for predicting response to ICIs. Nevertheless, this study is limited in establishing NMR as a valid predictive biomarker owing to the absence of a large-scale validation cohort. Our study demonstrated that the anti-PD1 response of NSCLC patients was related to on-treatment NMR, and not the baseline NMR. Therefore, this is a proof-of-concept study, and thus careful interpretation is needed until confirmation is obtained. However, the baseline characteristics of patients in our study is different from those of the Checkmate 017 or 057 and the number of patients is small. Therefore, we need to cautiously interpret our result as surrogate markers for predicting response in NSCLC patients treated with PD-1 blockade.

In conclusion, the ratio of NK cells to Lox-1^+^ PMN-MDSCs in the peripheral blood mononuclear cells (PBMCs) of patients with NSCLC after the first round of nivolumab treatment was strongly correlated with treatment outcomes. NMR measured with blood samples might provide clinicians with more accurate information as to whether to continue or stop further treatment and consequently improve the benefit of anti-PD-1 therapy for NSCLC patients.

## Methods

### Study population, treatment, and outcomes

We prospectively enrolled patients with NSCLC who had failed platinum-based chemotherapy between April 2015 and February 2018 at Yonsei Cancer Centre in Seoul, Korea. We conducted this study in accordance with the principles of the Declaration of Helsinki and Good Clinical Practice. The study was approved by the institutional review board of Severance Hospital. All patients provided written informed consent. The four inclusion criteria were: >20 years of age, confirmed diagnosis of recurrent/metastatic NSCLC, failed platinum-based chemotherapy, received at least one cycle of nivolumab (3 mg/kg every 2 weeks), and willingness to provide informed consent. Exclusion criteria were the presence of autoimmune disease, active viral infection (HIV, HBV, or HCV), pregnancy, symptomatic brain metastases, or the receipt of concomitant systemic therapy for NSCLC.

Treatment efficacy was assessed using contrast-enhanced computed tomography at approximately 8 weeks after the first nivolumab infusion; clinical responses were classified as complete response (CR), partial response (PR), stable disease (SD), or progressive disease (PD) based on the Response Evaluation Criteria in Solid Tumours, version 1.1^24^. The objective response rate (ORR) was defined as the summation of PR and CR. Responders were defined as those classified as CR, PR, or SD for more than 6 months, and non-responders were defined as those classified as PD or SD for less than 6 months. Some of the clinical data have been reported in our prior study^13^. Tumor PD-L1 expression was determined immunohistochemically using the PD-L1 22C3 pharmDx antibody (Dako North America Inc., Carpinteria, CA, USA) or Ventana PD-L1 SP263 antibody (Ventana Medical Systems, Tucson, AZ, USA) as companion diagnosis.

### Quantification of immune cells in peripheral blood

Peripheral blood was collected within 5 days before the first nivolumab treatment (point 1, baseline) and at 0 to 3 days before the second treatment (point 2, after the first infusion). Blood samples were collected with ethylenediaminetetraacetic acid. PBMCs were isolated by density-gradient centrifugation using Ficoll (GE Healthcare). Cells were stained with antibodies (Supplementary Table S1) and analysed using FACS LSR Fortessa (BD Biosciences) immediately after isolation (schematic in Fig. 1). Data were analysed with FlowJo software (Treestar).

### Statistical analysis

Statistical analyses were performed using the GraphPad Prism 5.0 software package (GraphPad Software, Inc.). Data are presented as the mean values ± standard deviations. *P*-values were calculated between groups (i.e., responders vs. non-responders) using Student’s *t*-test or with log-rank statistics for survival analyses. PFS was defined as the time between the initiation of anti-PD-1 therapy and progression to disease or any cause of death. OS was defined as the time between initiation of anti-PD-1 therapy and death from any cause. AHRs for the risk of progression or death in response to anti-PD-1 therapy according to the NMR cut-off value were calculated using a Cox regression model that included age, sex, smoking, and genetic alteration as independent variables. All *P*-values were based on a two-tailed hypothesis.

## Supplementary information

Supplementary Information.

Supplementary Information2.

Supplementary Information3.

## Data Availability

The datasets generated during and/or analysed during the current study are available from the corresponding author on reasonable request.
