# Supplementary material for: Peripheral natural killer cells and myeloid-derived suppressor cells correlate with anti-PD-1 responses in non-small cell lung cancer
Source: Sci Rep. 2020 Jun 3;10:9050. doi: 10.1038/s41598-020-65666-x (PMC7270107; doi:10.1038/s41598-020-65666-x)
Supplement: Supplementary file 1 — Supplementary Information. [file 41598_2020_65666_MOESM1_ESM.docx]

**Title: Peripheral natural killer cells and myeloid-derived suppressor cells correlate with anti-PD-1 responses in non-small cell lung cancer**

**Authors’ name and title**

Prof. Dr. Je-In Youn, PhD

Su-Myeong Park, MS

Seyeon Park, MS

Gamin Kim, MS

Hee-Jae Lee, MS

Dr. Jimin Son, PhD

Prof. Min Hee Hong, MD

Aziz Ghaderpour, MS

Bumseo Baik, MS

Jahirul Islam, MS

Ji-Woong Choi, PhD

Eun-Young Lee, MS

Hang-Rae Kim, PhD

Prof. Sang-Uk Seo, PhD

Prof. SOONMYUNG PAIK, MD, PhD

Prof. Hong In Yoon, MD, PhD

Prof. Inkyung Jung, PhD

Xin Chun-Feng, MS

Hyun-Tak Jin, PhD

Prof. Seung-Yong Seong, MD, PhD

Prof. Sang-Jun Ha, PhD

Prof. Hye Ryun Kim, MD, PhD

**Supplementary Table S1. Antibodies used for flow cytometry**

| **Specificity** | **Clone** | **Supplier** |
| --- | --- | --- |
| CD45 | HI30 | BD Biosciences |
| CD15 | HI98 | BD Biosciences |
| CD14 | MΦP9 | BD Biosciences |
| HLA-DR | G46-6 | BD Biosciences |
| CD56 | B159 | BD Biosciences |
| CD19 | SJ25C1 | BD Biosciences |
| CD3 | UCHT1 | BD Biosciences |
| CD33 | P67-6 | BD Biosciences |
| CD11b | ICRF44 | BD Biosciences |
| Lox-1 | 15C4 | BioLegend |
