# Supplementary figures and images for: Peripheral natural killer cells and myeloid-derived suppressor cells correlate with anti-PD-1 responses in non-small cell lung cancer
Source: Sci Rep. 2020 Jun 3;10:9050. doi: 10.1038/s41598-020-65666-x (PMC7270107; doi:10.1038/s41598-020-65666-x)

Supplementary Figure 1

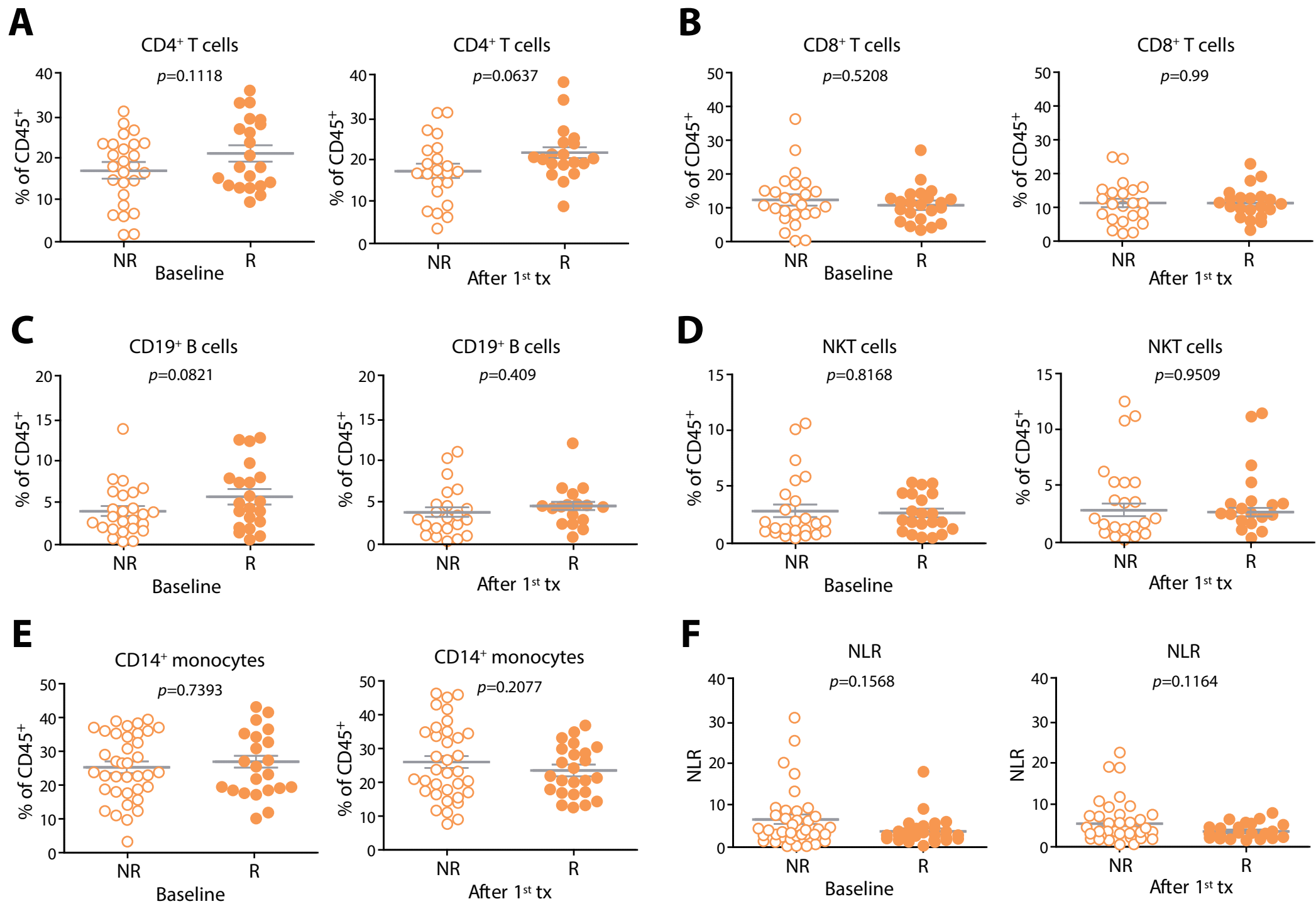

Supplement: Supplementary file 2 — Supplementary Information2. [file 41598_2020_65666_MOESM2_ESM.pdf]
