# Supplementary material for: Peripheral natural killer cells and myeloid-derived suppressor cells correlate with anti-PD-1 responses in non-small cell lung cancer
Source: Sci Rep. 2020 Jun 3;10:9050. doi: 10.1038/s41598-020-65666-x (PMC7270107; doi:10.1038/s41598-020-65666-x)
Supplement: Supplementary file 3 — Supplementary Information3. [file 41598_2020_65666_MOESM3_ESM.docx]

**Supplementary Fig 1.**

Frequencies of the various immune cells between responders and non-responders at baseline and after 1^st^ treatment.
